# Systemic adaptation of lipid metabolism in response to low- and high-fat diet in Nile tilapia (*Oreochromis niloticus*)

**DOI:** 10.14814/phy2.12485

**Published:** 2015-08-11

**Authors:** An-Yuan He, Li-Jun Ning, Li-Qiao Chen, Ya-Li Chen, Qi Xing, Jia-Min Li, Fang Qiao, Dong-Liang Li, Mei-Ling Zhang, Zhen-Yu Du

**Affiliations:** 1 Laboratory of Aquaculture Nutrition and Environmental Health (LANEH), School of Life Sciences, East China Normal UniversityShanghai, China; 2 Pediatric Translational Medicine Institute, Shanghai Jiao Tong University School of MedicineShanghai, China

**Keywords:** Dietary lipid level, lipid homeostasis, lipid metabolism, Nile tilapia, triglyceride

## Abstract

Natural selection endows animals with the abilities to store lipid when food is abundant and to synthesize lipid when it is limited. However, the relevant adaptive strategy of lipid metabolism has not been clearly elucidated in fish. This study examined the systemic metabolic strategies of Nile tilapia to maintain lipid homeostasis when fed with low- or high-fat diets. Three diets with different lipid contents (1%, 7%, and 13%) were formulated and fed to tilapias for 10 weeks. At the end of the feeding trial, the growth rate, hepatic somatic index, and the triglyceride (TG) contents of serum, liver, muscle, and adipose tissue were comparable among three groups, whereas the total body lipid contents and the mass of adipose tissue increased with the increased dietary lipid levels. Overall quantitative PCR, western blotting and transcriptomic assays indicated that the liver was the primary responding organ to low-fat (LF) diet feeding, and the elevated glycolysis and accelerated biosynthesis of fatty acids (FA) in the liver is likely to be the main strategies of tilapia toward LF intake. In contrast, excess ingested lipid was preferentially stored in adipose tissue through increasing the capability of FA uptake and TG synthesis. Increasing numbers, but not enlarging size, of adipocytes may be the main strategy of Nile tilapia responding to continuous high-fat (HF) diet feeding. This is the first study illuminating the systemic adaptation of lipid metabolism responding to LF or HF diet in fish, and our results shed new light on fish physiology.

## Introduction

The main biological functions of lipids include storing energy, signaling, and acting as structural components of cell membranes. Abnormal fluctuations of endogenous lipids or lipid metabolites can cause a number of diseases, particularly metabolic syndromes, in most vertebrates (Eckel et al. 2005). Therefore, maintaining lipid homeostasis is critical for an organism’s survival, health, and reproduction (Brasaemle 2007). In evolutionary history, animals have developed an accurate and complicated metabolic system to adapt to different nutritional states. In nature, food is not always available, thus when food is plentiful, a lot of eating occurs and properly storing the excess energy as lipid in body in an innoxious way is a survival strategy for most animals. In contrast, when food or dietary lipid is very limited, accelerating lipid biosynthesis to satisfy the physiological lipid requirement is also necessary.

In mammalian models, the adaptive lipid metabolism responding to different dietary lipid levels has been largely discussed, especially in the high-fat diet (HF diet) feeding conditions that mimic the western dietary pattern of humans (Ikemoto et al. 1996; Lin et al. 2000; Buettner et al. 2007; Kohsaka et al. 2007). In general, mammals mostly store excess energy in the form of neutral lipid (triglyceride, TG) in white adipose tissue (WAT), a specific lipid deposit organ mainly composed of adipocytes. However, a sustained HF diet induces impaired lipid homeostasis, in which large amounts of lipid also pathologically accumulate in other organs such as the liver (Du et al. 2013). In lipid deficiency, de novo lipid biosynthesis will be accelerated, but the major sites of lipid biosynthesis are species-specific. For example, the liver and WAT are two main sources for newly synthesized lipid in rodents (Gondret et al. 2001; González et al. 2014), but lipogenesis in pigs occurs principally in WAT, whereas in avian species the liver is the main lipogenic site (Gondret et al. 2001). Along with lipid deposits and lipogenesis, lipid transport and lipid catabolism, mainly including lipolysis and fatty acid (FA) β-oxidation, also play important roles in maintaining lipid homeostasis (Jocken et al. 2007; Khasawneh et al. 2009). These metabolic pathways and organs constitute a fine lipid metabolic system with high adaptation toward different nutritional or physiological conditions and the metabolic imbalance between these pathways or organs leads to a series of metabolic syndromes, such as heart failure, obesity, diabetes, and non-alcoholic fatty liver disease in mammals (Unger et al. 2010).

Compared with mammals, fish, the largest vertebrate group, are poikilothermic and have lower capabilities to use carbohydrates as energy (Tocher 2003); thus, energy metabolism in fish is different from mammals. In nature, high energy intake and long-term food deficiency is also commonly seen in the life cycle of some fish, particularly migratory fish (Sandercock 1991). In addition, in modern aquaculture HF diets have been widely used in many economic fish species to save dietary protein as an energy source and increase feed efficiency (Hillestad et al. 1998; Boujard et al. 2004). However, HF diets commonly caused excess fat accumulation in the liver or visceral fat tissue in farmed fish, accompanied by low growth, survival and resistance to pathogens and environmental stresses (Regost et al. 2001; Wang et al. 2005a), suggesting impaired lipid homeostasis. So far, people have known that the liver, adipose tissues, or muscle have the capability to store lipid in fish (Lin et al. 1977; Ando et al. 1993; Kaneko et al. 2013), and a number of lipid-metabolism-related genes in some farmed fishes have been cloned and the preliminary functions have also been illustrated (He et al. 2014). However, at present, studies about the effects of dietary lipid on lipid metabolism in fish are mostly descriptive, or only focus on different organs separately. The lipid metabolism of fish, especially the adaptive capability to deal with different lipid intake, has not been well understood. Moreover, the lack of genomic information in most of farmed fish species limits the intensive study of the mechanisms of lipid metabolism in fish.

Nile tilapia (*Oreochromis niloticus*) is an important aquaculture species cultured worldwide. Compared with zebra fish (*Danio rerio*), Nile tilapia could be used as a better fish model for nutrition and metabolism studies, not only because it grows fast and has high resistance to diseases and toxic stress (Deng et al. 2010), but also its whole genomic information is available (Guyon et al. 2012). Moreover, Nile tilapia have well-developed digestive and metabolic organs, including liver, muscle, and adipose tissue. In this study, we used Nile tilapia as a fish model to systemically elucidate the metabolic strategies of fish to maintain lipid homeostasis when fed with low-fat (LF) or HF diets for 10 weeks. The results of histology, quantitative PCR (qPCR), metabolites content measurement, western blotting and transcriptomic assays indicated that the elevated glycolysis, accelerated biosynthesis of FA, and enhanced lipid transport mediated by lipoproteins in the liver were likely to be the main strategies of tilapia toward LF intake. In contrast, excess ingested lipid was preferentially stored in adipose tissue through increasing FA uptake and TG synthesis. Increasing numbers, but not enlarging size, of adipocytes was the main strategy of Nile tilapia responding to continuous HF diet feeding. This study provides a novel insight into the lipid nutrition and metabolism in fish.

## Materials and Methods

### Animals and feeding

Juvenile Nile tilapias weighed about 2 g were obtained from Shanghai Ocean University. The fishes were fed with a commercial diet (Dajiang, China) prior to experiment. Based on a number of previous nutritional studies in Nile tilapia, 5–7.4% was the optimal lipid level in diet of Nile tilapia (Santiago and Reyes 1993; Wang et al. 2005b; Abdel-Tawwab et al. 2010; Deng et al. 2010; Xiong et al. 2014). LF diet, medium fat (MF) diet, and HF diet (1%, 7% and 13% fat content, respectively) were formulated and coded as LF, MF, and HF, respectively (Table1). After 1-week acclimation, fishes were randomly divided into three dietary groups with three tanks (100 cm × 60 cm × 50 cm) per group and 30 fishes per tank. The daily feeding rate was 4% of fish biomass and the trial lasted for 10 weeks. During the trial, the photoperiod was 12 h daylight and 12 h nightlight. The temperature was maintained at 26 ± 2°C and the water was changed 1/3 every day. The body weights were weighed every 10 days and the exact feeding amounts were adjusted correspondently. After the trial, three individuals per tank were anesthetized using MS-222 after feeding for 1 h and dissected for liver, muscle, and visceral adipose tissue collection. Hepatic somatic index (HSI) and visceral adipose tissue somatic index (VSI) were calculated. HSI = the weight of liver/body weight (%) and VSI = the weight of visceral adipose tissue/body weight (%). All samples were stored at −80°C. All experiments were conducted under the Guidance of the Care and Use of Laboratory Animals in China. This research was approved by the Committee on the Ethics of Animal Experiments of East China Normal University.

**Table 1:** The formulation of the experimental diets

Component (g/kg)	LF	MF	HF
Casein	360	360	360
Gelatin	80	80	80
Soybean oil	10	70	130
Corn starch	324.75	324.75	324.75
Vitamin premix*	10	10	10
Mineral premix†	40	40	40
CMC	30	30	30
Cellulose	140	80	20
Choline chloride	5	5	5
BHT	0.25	0.25	0.25
Total	1000	1000	1000
Composition			
Dry matter (%)	92.31	92.27	92.25
Crude protein (%)	43.21	43.16	43.25
Crude lipid (%)	1.17	6.98	13.11
Ash (%)	5.23	5.19	5.16

* Mineral premix (g/kg): 314.0 g CaCO_3_; 469.3 KH_2_PO_4_; 147.4 g MgSO_4_·7H_2_O; 49.8 g NaCl; 10.9 g Fe(II) gluconate; 3.12 g MnSO_4_·H_2_O; 4.67 g ZnSO_4_·7H_2_O; 0.62 g CuSO_4_·5H_2_O; 0.16 g KJ; 0.08 g CoCl_2_·6H_2_O; 0.06 g NH_4_ molybdate; 0.02 g NaSeO_3_.

† Vitamin premix (mg or IU/kg): 500,000 I.U. (international units) Vitamin A, 50,000 I.U. Vitamin D3, 2500 mg Vitamin E, 1000 mg Vitamin K3, 5000 mg Vitamin B1, 5000 mg Vitamin B2, 5000 mg Vitamin B6, 5000 μg Vitamin B12, 25,000 mg Inositol, 10,000 mg Pantothenic acid, 100,000 mg Cholin, 25,000 mg Niacin, 1000 mg Folic acid, 250 mg Biotin, 10,000 mg Vitamin C.

### Histology and metabolites content measurement

The paraffin sectioning and hematoxylin and eosin staining of visceral adipose tissue were performed as described (Du et al. 2011). Two blocks of tissue per fish were sectioned and 10 sections per tissue were evaluated. Twelve sections per group were evaluated for counting cell number. The relative cell size (RAS, RAS = 1/cell number per unit square) was calculated. The relative total adipocyte number (RAN) = cell number per unit square × adipose tissue weight/body weight (assuming the density of adipose tissue is homogeneous). The TG content and aspartate transaminase (AST) activity were measured using commercial kits (Jiancheng Bioengineering Institute, Nanjing, China) performed as described in the protocols attached in kits. Lipid content in whole body was measured in Soxtec 2050 (FOSS Analytical AB, Höganäs, Sweden) using the standard protocol. RAS, RAN, and TG level were normalized using the data of LF group.

### Antibody and western blotting assay

Frozen tissues were homogenized on ice with a homogenizer in RIPA buffer from Beyotime (Catalog no. P0013B; Haimen City, Jiangsu, China). This buffer contained 50 mmol/L Tris (pH 7.4), 150 mmol/L NaCl, 1% Triton X-100, 1% sodium deoxycholate, 0.1% SDS, 1 mmol/L PMSF, and protease inhibitors. Homogenates were centrifuged for 10 min at 12,000 *g*. The supernatants were aliquoted and stored at −80°C. Protein concentrations were determined using the Beyotime protein assay kit (Catalog no. P0010). Cell lysates (45 μg of protein) were subjected to SDS-PAGE using the appropriate antibody. β-actin amino acid (AA) sequences of Nile tilapia were totally identical with those of mouse. The rabbit polyclonal antibody against mouse β-actin was obtained from HuaAn Biotech (Catalog no. R1102-1; Hangzhou, Zhejiang, China). Preliminary experiment was conducted to choose an appropriate antibody against peroxisome proliferator-activated receptor gamma (PPARγ). Finally, a rabbit polyclonal antibody against mouse PPARγ from Proteintech (Catalog no. 16643-1-AP; Proteintech Group, Inc., Chicago, IL) was chosen. The AA sequence of mouse PPARγ used to produce this antibody was 318 AA in length and had 75.0% identity with the correspondent sequence of PPARγ in Nile tilapia. IRDye 800CW goat (polyclonal) anti-Rabbit secondary antibody was from LI-COR (Catalog no. 926-32211; LI-COR Biosciences Corporate, Lincoln, NE). Bands were visualized by infrared fluorescence using the Odyssey imaging system (LI-COR Biotechnology).

### RNA extraction and quantitative real-time PCR

RNA isolation, cDNA synthesis, and qPCR was performed as described previously (He et al. 2014). Briefly, 800 ng of total RNA was used to synthesize cDNA by using random primers. All primer pairs were designed to overlap one intron to avoid amplification of genomic DNA (the elongation time was limited so that the PCR using genomic DNA as template failed). The products of amplification were cloned into pMD19-T vector to sequencing for confirming the accuracy of amplicon (the lengths were ranged from 92 to 112 bp). A series diluted cDNA was used to assess the amplification efficiency. The primer pairs with the efficiency of amplification between 99% and 101% were chose to conduct qPCR (Table2). Two genes stably expressed in different dietary groups, β-actin and elongation factor 1 alpha (EF1α), were chosen for qPCR normalization. The methods 2^−ΔΔCt^ was used (LF group as the control), and values of three groups were further normalized by fixing LF group as 1. Every target gene measured and the normalized genes in each cDNA sample were always determined on the same plate. The qPCR was performed on CFX Connect Real-Time System (Bio-Rad Laboratories, Inc., Hercules, CA).

**Table 2:** The primers used in this study

Function classifications	Gene name	Sense and antisense primer (5′–3′)	Size (bp)	GenBank No.
Internal reference	β-actin	CAGGATGCAGAAGGAGATCACA	92	KJ126772
		CGATCCAGACGGAGTATTTACG		
	Elongation factor 1 alpha (EF1α)	CTACGTGACCATCATTGATGCC	106	KJ123689
		AACACCAGCAGCAACGATCA		
Regulatory factors	Peroxisome proliferator-activated receptor alpha (PPARα)	CTGATAAAGCTTCGGGCTTCCA	106	KF871430
		CGCTCACACTTATCATACTCCAGCT		
	Peroxisome proliferator-activated receptor beta (PPARβ)	CAGGAGAACAGTGACAACAAGCA	100	KF751705
		CCAAACGCAGCTGTTCTGAGA		
	Peroxisome proliferator-activated receptor gamma variant 1 (PPARγ1)	TCCAGCTTCAGAAACAGAGAGTGTG	111	KF918712
		AGACTGAAGCCAACAGGCCA		
	Peroxisome proliferator-activated receptor gamma variant 2 (PPARγ2)	CAGGCAGAGATTTTACCCATCAAAC	104	KF918713
		TGCTGTGTTCAGACTGAAGCCAA		
	Sterol regulatory element-binding transcription factor 1 (SREBP1)	TGCAGCAGAGAGACTGTATCCGA	102	XM_005457771
		ACTGCCCTGAATGTGTTCAGACA		
Lipid uptake	Fatty acid transport protein 5 (FATP5)	TACACATCTGGGACCACAGGTTTG	110	XM_003443859.2
		AAGATGTCCTCTGCTGTGACTCCA		
	Cluster determinant 36 (CD36)	TGGAGCACTGGACATCAGTTCCT	96	XM_003452029
		CCCAACACAACCTCCCGTAGATAT		
	Hepatic lipase (HL)	GCAGACGCTACAGGAGCACTACAA	97	FJ436083.1
		AAAGCTCCCAGCAAATCCAGAGAT		
	Lipoprotein lipase (LPL)	CACCAAACTAGTGGGTCGTGATGT	103	NM_001279753
		TCCCAGACTATAACCCAGCAGATGA		
Intracellular fatty acid transportation	Fatty acid-binding protein 4 (FABP4)	AAGCTGGGAGAGGAGTTTGATGAA	112	XM_003458335
		TCTCTTTGCCGTCCCACTTCT		
Fatty acid synthesis	Acetyl-CoA carboxylase alpha (ACCα)	TAGCTGAAGAGGAGGGTGCAAGA	110	XM_005471970
		AACCTCTGGATTGGCTTGAACA		
	ATP citrate lyase (ACLY)	AAAAGCTTTGATGAGCTTGGGG	102	XM_003442027
		TACAGTGGGAGGAGGCAACTCTT		
	Fatty acid synthase (FAS)	TCATCCAGCAGTTCACTGGCATT	102	GU433188
		TGATTAGGTCCACGGCCACA		
Glycerol synthesis	Phosphoenolpyruvate carboxykinase 1 (PEPCK1)	TGGAAGAACAAACCTTGGCG	99	XM_003448375
		TGGGTCAATAATGGGACACTGTCT		
TG synthesis	Glycerol-3-phosphate acyltransferase 1 (GPAT)	ATAACATCAAAGCCCCGCACAT	105	XM_005471309
		CCATTCTTCGTCGTATGAAGAAACC		
	Diacylglycerol O-acyltransferase 2 (DGAT2)	GCTTGAATTCTGTCACCCTGAAGA	106	XM_003458972
		ACCTGCTTGTAGGCGTCGTTCT		
Lipolysis	Hormone-sensitive lipase (HSL)	AACCTGGATGTCCATTTCTGGAAG	102	FJ601660
		TCGGTTTACCTTGACTTGAGTGGA		
	Adipose triglyceride lipase (ATGL)	AAAACGTCCTGGTGACCCAGT	104	XM_003440346
		TAGGAGGAATGATGCCACAGTACA		
Fatty acid β-oxidation	Acetyl-CoA carboxylase beta (ACCβ)	ACATGCAGTCCATGCTGCGT	106	XM_003451659
		AAATGCCTCTCAAGCCACTCAA		
	Carnitine palmitoyltransferase I alpha (CPT1a)	TTTCCAGGCCTCCTTACCCA	102	XM_003440552
		TTGTACTGCTCATTGTCCAGCAGA		
	Carnitine palmitoyltransferase I beta (CPT1b)	AAGGGACGTTACTTCAAGGTG	101	GQ395696
		TCCGACTTGTCTGCCAAGAT		
Very low-density lipoprotein protein	ApoB	TCCCCAGCTACACTGCACAGTT	102	XM_005461940
		CATCGCCTCTTCCTGACATCATC		
	ApoE	ATAAGCTGCAGAAGCGCCTCAATA	106	XM_003447249
		TTCACTGTATCCAGGTTCTGGGAG		

The genes investigated in this study were related to: metabolic regulatory factors (PPARα, PPARβ, PPARγ1, PPARγ2, and sterol regulatory element binding protein 1 [SREBP1]), PPARγ1 and PPARγ2 were only different at 5′-untranslated region of mRNA; FA uptake (hepatic lipase [HL], lipoprotein lipase [LPL], fatty acid transport protein 5 [FATP5], Cluster of Differentiation 36 [CD36, also known as FA translocase]); intracellular FA transportation (fatty acid binding protein 4 [FABP4]); FA biosynthesis de novo (fatty acid synthase [FAS], ATP-citrate lyase [ACLY], and acetyl-CoA carboxylase alpha [ACCα]); fatty acid β-oxidation (Acetyl-CoA carboxylase beta [ACCβ], carnitine palmitoyl transferase 1 [CPT1a and CPT1b]); glyceroneogenesis (phosphoenolpyruvate carboxykinase 1 [PEPCK1]), triacylglycerol synthesis (glycerol phosphate acyltransferase [GPAT], and diacylglycerol O-acyltransferase 2 [DGAT2]); very low-density lipoprotein related protein (apoprotein B [ApoB] and apoprotein E [ApoE]), and lipolysis (hormone sensitive lipase [HSL] and adipose triglyceride lipase [ATGL]). The primers and estimated functions of all the genes measured were listed in Table2.

### Transcriptomic assay of liver

Three total hepatic RNAs per dietary group were pooled in equal amount. Pooled RNA of 5 μg was used to isolate mRNA by using magnetic beads (Invitrogen Corporation, Carlsbad, CA). RNA Sequencing Library Preparation and Deep Sequencing were performed as described (Li et al. 2013). In this study, each sample was subjected to 200 cycles of sequencing from both ends in one lane of an Illumina Hiseq2000 Sequencer (Illumina, Inc., San Diego, CA). For each sample, reads with a quality score of >Q20 that passed filtering were used to generate a complete FASTQ file, which was then mapped to UCSC Nile tilapia reference (Jan. 2011 [Broad oreNil1.1/oreNil2]) using Tophat2 (v2.0.2) (Kim et al. 2013) with the default parameter setting of 40 alignments per read and up to 2 mismatches per alignment. The resulting aligned reads were then analyzed with Cufflinks suite (v2.1.1) (http://cufflinks.cbcb.umd.edu) which assembles the aligned reads into transcripts and measures their relative abundance. The expression level of transcripts was assessed using FPKM (fragments per kilobase of exon per million fragments mapped). On the basis of the *P* values, the false discovery rate (FDR) was calculated by the method of Benjamini and Hochberg (1995), and the change between two groups was viewed as significant when FDR ≤ 0.05, ¦logFC¦ ≥ 1. Raw reads were deposited at the National Center for Biotechnology Information’s Sequence Read Archive under accession no. SRX838027 (LF, DL; MF, ZL; and HF, GL). Cluster analysis was conducted using distance algorithm (Spearman between samples and Pearson between genes). GO (Gene Ontology) enrichment analysis was conducted by using Goatools (https://github.com/tanghaibao/GOatools). Bonferroni, Holm, Sidak, and FDR were used to control the rate of false positive.

### Statistical analysis

All data are presented as mean ± SEM. Significant differences (*P* < 0.05) of each variable were first determined using the one-way analyses of variance test, followed by Tukey–Kramer test to rank the three experimental groups. All analyses were performed using SPSS 19.0 software (SPSS, Chicago, IL).

## Results

### Growth performance and body lipid content

**Figure 1:**
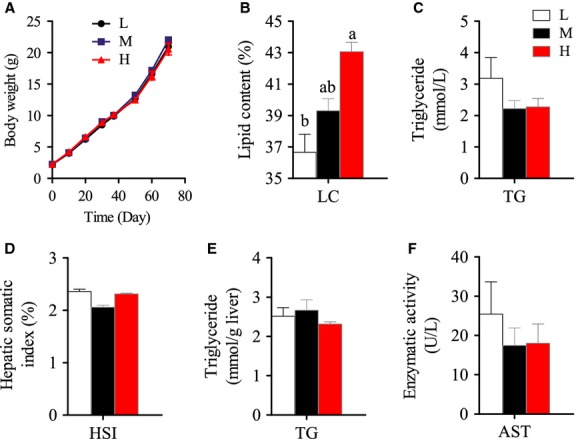
Growth, body lipid content, hepatic somatic index, triglyceride content in serum and liver, and aspartate aminotransferase activity in serum of Nile tilapia fed with diets containing low (1%), medium (7%), and high (13%) lipid content for 10 weeks. (A) Growth curve of Nile tilapia during 10-week feeding trial. (B) Crude lipid content in whole body (dry matter) of Nile tilapia at the end of the feeding trial. (C) Serum triglyceride (TG) content. (D) Hepatic-somatic index (HSI). (E) Hepatic TG content. (F) Serum aspartate transaminase (AST) activity. For A and B, Values are means ± SEM (*n* = 3); for C–F, Values are means ± SEM (*n* = 6). Values with different letters on columns statistically differ at *P *<* *0.05.

In this study, the tilapia grew from 2 g to more than 20 g during 10 weeks. At the end of the trial, there were no significant differences in final body weight among three dietary groups (Fig.1A). This was in accordance with a previous study in which dietary lipid content ranged from 0% to 15% did not significantly affect final body weights of juvenile tilapia in 8 weeks (Chou and Shiau 1996). This verified that our experimental fish and feeding conditions were normal. As predicted, total body lipid contents of the experimental fish significantly increased with increased dietary lipid content from 1% to 13% (Fig.1B); however, serum TG concentrations were comparable among the three groups (Fig.1C). This suggests that in the present experiment tilapia stored excess lipid intake in the body but still maintained lipid homeostasis.

### Liver is the main site of lipid biosynthesis de novo in Nile tilapia when fed with LF diet

**Figure 2:**
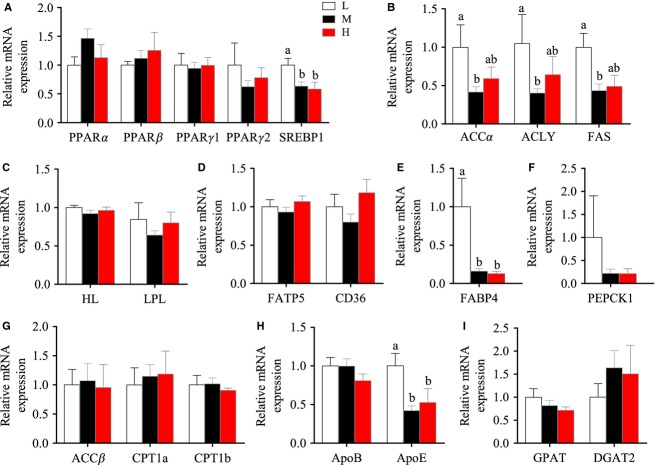
The mRNA expression of the genes in hepatic triglyceride (TG) metabolism of Nile tilapia fed with diets containing low (1%), medium (7%), and high (13%) lipid content for 10 weeks. (A) The relative mRNA abundance of PPARα, PPARβ, PPARγ1, PPARγ2, and SREBP1 in liver. (B) The relative mRNA abundance of ACCα, ACLY, and FAS showing the FA biosynthetic activity de novo. (C) The relative mRNA abundance of HL and LPL showing the ability of triglyceride hydrolysis. (D) The relative mRNA abundance of FATP5 and CD36 showing the ability of FA uptake. (E) The relative mRNA abundance of FABP4 showing the activity of intracellular FA transport. (F) The relative mRNA abundance of PEPCK1 participating in the pathway of glyceroneogenesis. (G) The relative mRNA abundance of ACCβ, CPT1a, and CPT1b showing the activity of FA β-oxidation. (H) The relative mRNA abundance of ApoB and ApoE which are important components of VLDL. (I) GPAT and DGAT2 showing the activity of TG synthesis. All values are means ± SEM (*n* = 6). Values with different letters on columns statistically differ at *P *<* *0.05.

There were no significant changes of HSI and hepatic TG contents among the three groups (Figs.1D and E). The activity of serum AST, a marker of hepatic injury, was also not altered by dietary lipid levels (Fig.1F). Considering the dietary lipid levels varied from 1% to 13%, the comparable hepatic lipid contents in the three groups suggested that Nile tilapia had adaptive mechanisms to maintain lipid homeostasis in the liver. Thus, we further examined the hepatic mRNA levels of several important metabolic regulatory factors involved in lipid metabolism (PPARα, PPARβ, PPARγ1, PPARγ2, and SREBP1) and found that only SREBP1 was significantly higher in the LF group than the MF and HF groups (Fig.2A). Higher SREBP1, a master regulator of lipogenesis, in the LF group led us to speculate that lipogenesis was increased in the liver when lipid intake was limited. Compared with the MF and HF groups, the higher expression of lipogenic genes (ACCα, ACLY, and FAS), along with the comparable expression of exogenous FA transport genes (LPL, HL, FATP5, and CD36) in the LF group confirmed our speculation (Fig.2B–D). This suggests that FA synthesis de novo was likely to contribute more to hepatic FA pool in LF group. Moreover, higher expression of FABP4, an important binding protein in intracellular FA transport (Furuhashi et al. 2014), in the LF group suggested increased intracellular FA metabolism (Fig.2E). However, the TG contents in the liver were comparable among the three groups (Fig.1E), showing higher FA synthesis did not cause higher TG accumulation in the LF group. This might because (1) the newly synthesized FAs were broken down through β-oxidation, or (2) the hepatic TGs biosynthesized in the LF group were transported by VLDL, or (3) the amount of the substrates for liver TG synthesis in LF group were comparable to those of other two groups. Thus we measured key genes in FA β-oxidation (ACCβ, CPT1a and CPT1b) (Fig.2G), but no significant differences were found among three groups. We also measured the mRNA levels of both ApoE and ApoB. The significantly higher expression of ApoE, but not ApoB, in the LF group versus the other two groups indicated that the altered lipoprotein transporting efficiency may help to maintain stable lipid contents in the liver (Fig.2H). We finally measured the genes in glycerol synthesis (PEPCK1) (Fig.2F) and triglyceride synthesis (GPAT and DGAT2) (Fig.2I), but no significant differences were found among three groups, suggesting the total amount of glycerol and FA – the substrates of TG synthesis were comparable among three groups, although FA de novo synthesis increased in the LF group. However, it must be noted that the posttranscription modification would also affect the amount of proteins coded by these genes. Compared with LF group, it was noticed that the MF and HF groups had similar metabolic and molecular characteristics in the liver, suggesting that the liver of tilapia is not sensitive to HF intake in a normal physiological state.

**Figure 3:**
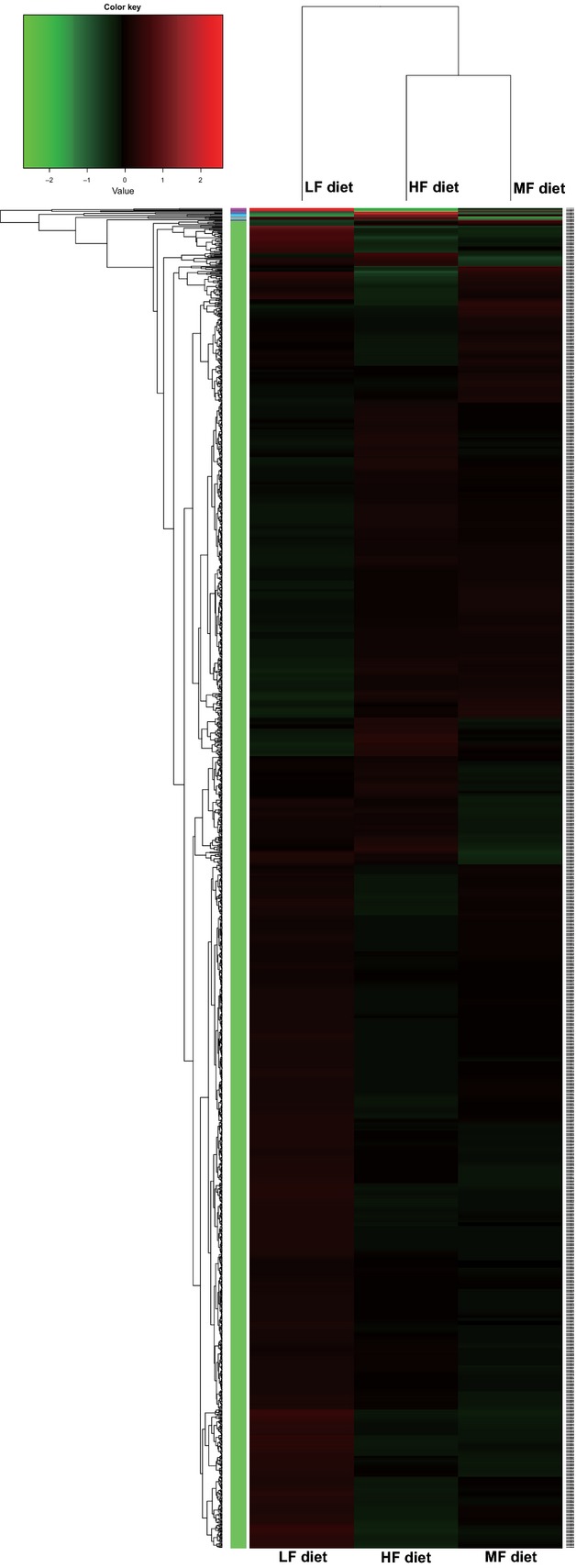
Clustered heat map of hepatic transcriptome of Nile tilapia fed with diets containing low (1%), medium (7%), and high (13%) lipid content for 10 weeks. Cluster analysis was conducted using distance algorithm (Spearman between samples and Pearson between genes). Transcript enrichment is encoded in the heat map from low (green) to high (red). Transcripts that show similar expression patterns are clustered together, as indicated by the colored groups to the left of the heat map.

To understand fully the physiological changes in the liver caused by different dietary lipid levels, we conducted transcriptomic analyses for liver samples by using Hiseq-2000. The number of high-quality reads (>Q20) was 44,096,624, 59,359,238, and 50,967,622 in the LF, MF, and HF groups, respectively. The reads were mapped on the genome of Nile tilapia and the mapping rates of the LF, MF, and HF groups were 85.64%, 84.35%, and 86.49%, respectively. Cluster analysis showed that the MF and HF groups were clustered together, and the LF was separated from MF and HF (Fig.3). This indicated that the liver of Nile tilapia had similar physiological responses to MF and HF diets, but had specific metabolic changes in the LF diet feeding. This was in accordance with the results obtained from real-time PCR (Fig.2A–I).

There was no enriched KEEG pathway found in any group pair (LF vs. MF, LF vs. HF, and MF vs. HF), but some enriched GO pathways were observed in three group pairs and these enriched pathways were rearranged and organized (Table3). There were 1004 differently expressed genes enriched in 46 pathways in LF versus MF, 1200 genes in 41 pathways in LF versus HF, and only 506 genes in 17 pathways in MF versus HF. These results corresponded to the findings in cluster analysis that the cluster distance between the MF and HF groups was closer than that between LF and MF or between LF and HF. FA metabolic processes, FA biosynthetic processes, lipid transport, and lipid biosynthetic processes, of which the key enzymes have been investigated by qPCR, were included in the enriched pathways. These results further confirmed the findings in liver using qPCR. Both of these findings suggested that hepatic lipogenesis in the LF group increased to cope with the deficiency of dietary lipids, whereas higher dietary lipid intake would not cause significant metabolic alterations in the liver of Nile tilapia.

**Table 3:** Gene ontology analysis of hepatic transcriptome

GO id	Description	Ratio in study		
		LF versus HF	LF versus MF	MF versus HF
GO:0006629	Lipid metabolic process	42/1200	34/1004	22/506
GO:0006631	Fatty acid metabolic process	11/1200	10/1004	
GO:0006633	Fatty acid biosynthetic process	9/1200	9/1004	
GO:0008610	Lipid biosynthetic process	26/1200	22/1004	
GO:0006869	Lipid transport		13/1004	
GO:0003824	Catalytic activity	347/1200	327/1004	
GO:0003987	Acetate-CoA ligase activity	3/1200	3/1004	
GO:0004497	Monooxygenase activity	15/1200	15/1004	10/506
GO:0005506	Iron ion binding	29/1200	26/1004	16/506
GO:0005783	Endoplasmic reticulum	20/1200	12/1004	8/506
GO:0005996	Monosaccharide metabolic process	14/1200	15/1004	
GO:0006006	Glucose metabolic process	11/1200	11/1004	
GO:0006082	Organic acid metabolic process	32/1200	28/1004	
GO:0006083	Acetate metabolic process	3/1200	3/1004	
GO:0006085	Acetyl-CoA biosynthetic process	4/1200	3/1004	
GO:0006091	Generation of precursor metabolites and energy	11/1200	10/1004	
GO:0006096	Glycolysis		8/1004	
GO:0007050	Cell cycle arrest	6/1200		
GO:0008150	Biological_process	673/1200	585/1004	
GO:0008152	Metabolic process	349/1200	322/1004	
GO:0008443	Phosphofructokinase activity	6/1200		
GO:0009055	Electron carrier activity	21/1200	17/1004	11/506
GO:0009628	Response to abiotic stimulus	12/1200		
GO:0016208	AMP binding	3/1200	3/1004	
GO:0016405	CoA-ligase activity	5/1200	5/1004	
GO:0016418	S-acetyltransferase activity			2/506
GO:0016491	Oxidoreductase activity	65/1200	65/1004	30/506
GO:0016705	Oxidoreductase activity, acting on paired donors, with incorporation or reduction of molecular oxygen	24/1200	25/1004	17/506
GO:0016746	Transferase activity, transferring acyl groups			12/506
GO:0016877	Ligase activity, forming carbon-sulfur bonds	6/1200	6/1004	
GO:0016878	Acid-thiol ligase activity	6/1200	6/1004	
GO:0019200	Carbohydrate kinase activity	7/1200	7/1004	
GO:0019318	Hexose metabolic process	14/1200	15/1004	
GO:0019427	Acetyl-CoA biosynthetic process from acetate	3/1200	3/1004	
GO:0019752	Carboxylic acid metabolic process	31/1200	27/1004	
GO:0019842	Vitamin binding			8/506
GO:0020037	Heme binding	22/1200	20/1004	11/506
GO:0031406	Carboxylic acid binding			6/506
GO:0031418	L-ascorbic acid binding		6/1004	5/506
GO:0032787	Monocarboxylic acid metabolic process	15/1200	14/1004	
GO:0035384	Thioester biosynthetic process	4/1200		
GO:0043436	Oxoacid metabolic process	32/1200	28/1004	
GO:0044283	Small molecule biosynthetic process		15/1004	
GO:0044710	Single-organism metabolic process		297/1004	
GO:0044711	Single-organism biosynthetic process		15/1004	
GO:0045786	Negative regulation of cell cycle	6/1200		
GO:0046394	Carboxylic acid biosynthetic process		15/1004	
GO:0046906	Tetrapyrrole binding	23/1200	20/1004	13/506
GO:0046914	Transition metal ion binding	32/1200	31/1004	17/506
GO:0047150	Betaine-homocysteine S-methyltransferase activity		3/1004	
GO:0048029	Monosaccharide binding		6/1004	5/506
GO:0055114	Oxidation-reduction process	63/1200	64/1004	29/506
GO:0071616	Acyl-CoA biosynthetic process	4/1200		
GO:0072330	Monocarboxylic acid biosynthetic process	9/1200	10/1004	

### Adipocyte proliferation is the main strategy in Nile tilapia responding to HF intake

**Figure 4:**
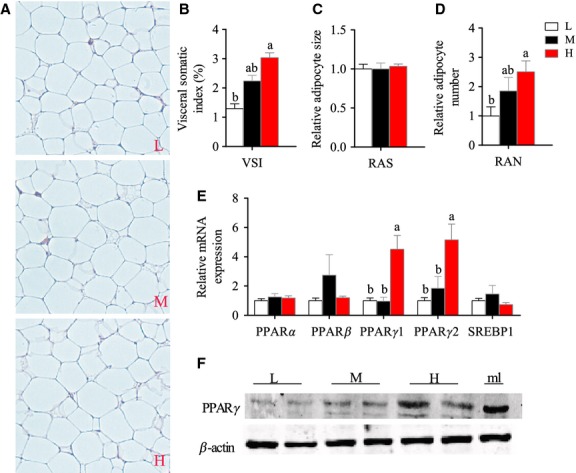
Histological characteristics and expression of regulatory genes related to adipocyte proliferation of Nile tilapia fed with diets containing low (1%), medium (7%), and high (13%) lipid content for 10 weeks. (A) Representative histologic appearance of visceral adipose tissue. (B) Visceral adipose tissue-somatic index (VSI). (C) Relative adipocyte size. (D) Relative visceral adipocyte number in whole adipose tissue. (E) The relative mRNA abundance of PPARα, PPARβ, PPARγ1, PPARγ2, and SREBP1 in visceral adipose tissue. (F) The result of western blotting, PPARγ antibody was used (top) and β-actin antibody was used as a loading control (bottom), a protein sample from mouse liver was loaded as positive control – the lane (mL). For B–E, values are means ± SEM (*n* = 6). Values with different letters on columns statistically differ at *P *<* *0.05.

The mass of visceral adipose tissue, which was presented by VSI, increased with the increased dietary lipid level in tilapia (Fig.4B). The histological images of adipose tissues are shown in Figure4A. By calculating the size and number of adipocytes, we found the increased adipose tissue mass was mainly caused by increased adipocyte numbers (Fig.4D), but not the enlargement of the size of adipocytes (Fig.4C). PPARs and SREBP1 are the important nuclear receptors in adipogenesis (Eberle et al. 2004; Wang 2010), whereas the mRNA expression of these genes showed only PPARγ significantly highly expressed in the HF group (Fig.4E). Moreover, western blotting also verified that the protein levels of PPARγ in adipose tissue increased with increasing dietary lipid levels (Fig.4F). Because PPARγ has been shown to be a critical receptor in regulating the differentiation and multiplication of adipocytes in mammals and fishes (Semple et al. 2006; Bouraoui et al. 2008), the higher mRNA and protein levels of PPARγ in the HF group confirmed again that the proliferation of adipocytes is likely to be the main strategy of Nile tilapia responding to HF intake.

**Figure 5:**
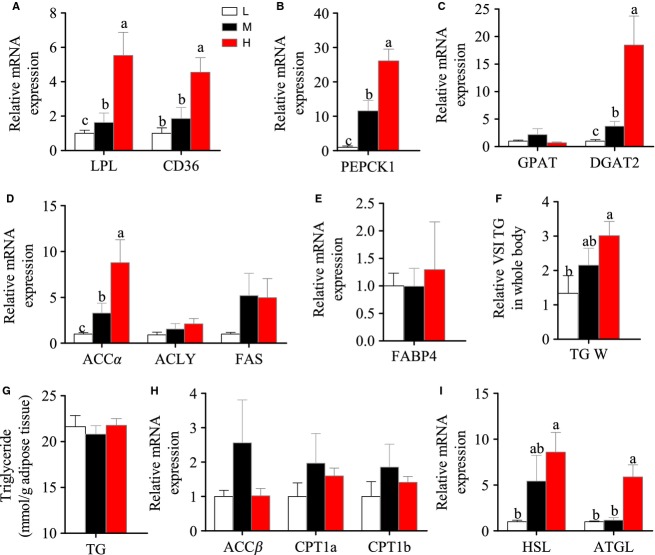
The mRNA expression of the genes in TG metabolism in adipose tissue of Nile tilapia fed with diets containing low (1%), medium (7%), and high (13%) lipid content for 10 weeks. (A) The relative mRNA abundance of CD36 and LPL showing the ability of FA uptake. (B) The relative mRNA abundance of PEPCK1 participating in the pathway of glyceroneogenesis. (C) The relative mRNA abundance of GPAT and DGAT2 showing the activity of TG synthesis. (D) The relative mRNA abundance of ACCα, ACLY, and FAS showing the FA biosynthetic activity de novo. (E) The relative mRNA abundance of FABP4 showing the activity of intracellular FA transport. (F) The relative visceral adipose TG level. (G) The relative visceral adipose TG level in whole body (right). (H) The relative mRNA abundance of ACCβ,CPT1a, and CPT1b showing the activity of FA β-oxidation. (I) The relative mRNA abundance of HSL and ATGL showing the activities of lipolysis. For A–I, values are means ± SEM (*n* = 6). Values with different letters on columns statistically differ at *P *<* *0.05.

To understand the metabolic details in adipose tissue of Nile tilapia in the HF group, a number of lipid metabolism-related genes were measured. Compared with the LF and MF groups, the higher mRNA expression of the genes related to exogenous FA uptake (CD36 and LPL) (Fig.5A), glycerol synthesis (PEPCK1) (Fig.5B), and TG synthesis (DGAT) (Fig.5C) in the HF group clearly indicated that adipose tissue could efficiently uptake excess exogenous FFA and store it in the form of TG. Of note, the genes related to intracellular FA biosynthesis, such as ACCα and FAS (Fig.5D), also expressed higher in HF group, suggesting some newly synthesized FAs could also contribute to the TG synthesis. Although the total adipose tissue mass and the total TG contents in whole adipose tissue were significantly higher in the HF group than in the other two groups (Fig.5F), we noticed that the adipocyte size and the TG contents per unit weight of adipose tissue were similar in the three groups (Figs.4C and 5G). This suggested that the lipid contents in a single adipocyte of tilapia are accurately regulated and excess cellular lipid would be decreased in some way. Thus, we measured the mRNA levels of three enzymes involved in FA β-oxidation (ACCβ, CPT1a, and CPT1b) (Fig.5H), which could break down the intracellular FFA, and two enzymes that are responsible for lipolysis of TG (HSL and ATGL) (Fig.5I). The dietary lipid content-dependent increase in mRNA levels was only seen in lipolytic genes. This indicated that the excess TG in adipocytes would be lipolyzed and the released FFA would be absorbed by newly differentiated adipocytes. In fact, some studies have reported that high FFA would upregulate the expression of PPARγ and accelerate adipocyte proliferation (Yessoufou and Wahli 2010).

### Lipid metabolism in muscle

**Figure 6:**
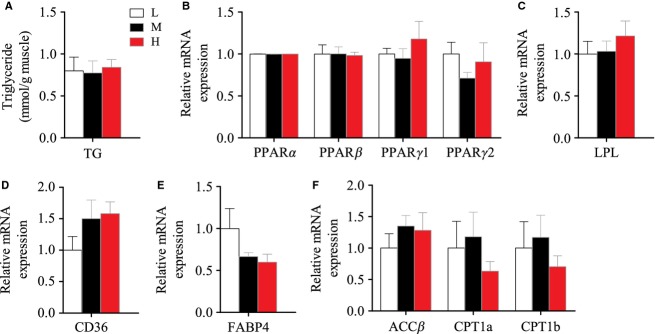
The mRNA expression of the genes in TG metabolism in muscle of Nile tilapia fed with diets containing low (1%), medium (7%), and high (13%) lipid content for 10 weeks. (A) The relative abundance of TG. (B) The relative mRNA abundance of PPARα, PPARβ, PPARγ1, and PPARγ2. (C) The relative mRNA abundance of LPL. (D) The relative mRNA abundance of CD36 showing the ability of FA uptake. (E) The relative mRNA abundance of FABP4 showing the activity of intracellular FA transport. (F) ACCβ, CPT1a, and CPT1b showing the activity of FA β-oxidation. For A–F, values are means ± SEM (*n* = 6). Values with different letters on columns statistically differ at *P *<* *0.05.

Different dietary lipid contents did not affect TG contents in muscle (Fig.6A). The mRNA levels of three lipid metabolic regulators (PPARα, PPARβ, and PPARγ) were comparable among the three groups (Fig.6B). Moreover, a number of genes related to FA uptake (Fig.6C and D), FA intracellular transport (Fig.6E), and FA β-oxidation (Fig.6F), which contribute to maintain TG homeostasis, did not change. The data of other lipogenic genes in muscle, including FA synthesis, glyceroneogenesis, and TG synthesis, were not presented because of the extremely low expression. These results indicated that the muscle of tilapia is unlikely to be the main responding organ to deal with high or low lipid intake.

## Discussion

### The strategy of Nile tilapia responding to low lipid intake

Dietary lipid (mainly TG) is important for almost all organisms, not only as a provider of essential FA (EFA) but also an important source of energy and signaling molecules. Therefore, limited dietary lipid is a nutritional stress for organisms. In this study, one of our purposes was to understand the metabolic strategies of tilapia to deal with LF intake. In many fish species, limited dietary lipid resulted in low growth and a series of symptoms related to EFA efficiency (Takeuchi et al. 1990; Watanabe 1993). However, some fish, especially herbivorous and omnivorous species, such as grass carp (*Ctenopharyngodon idella*) and tilapia, have the ability to endure EFA deficiency for a relatively long period and synthesize lipid when dietary lipid is low (Chou and Shiau 1996; Du et al. 2005). In grass carp, the optimal dietary lipid content was 3–6%, and diets containing 0%, 2%, and 4% lipid did not cause significant changes in growth, liver lipid content and mesenteric fat mass during a 10-week trial (Du et al. 2005). Similarly, there were no significant differences in body lipid content between the tilapia fed with the diets of 0% and 5% lipid contents at least during an 8-week laboratory trial (Chou and Shiau 1996). These results indicated that some fish could have the ability to adapt to LF intake by synthesizing lipid to promote endogenous lipid homeostasis, at least in a given period. However, few studies focused on the lipogenic sites and the systemic metabolic strategies of fish responding to LF intake. Even though a few studies had investigated some metabolic changes in the fish fed with LF diet, many contradictions were found in these preliminary works. In turbot (*Psetta maxima*), only hepatic lipogenic enzymes (glucose-6-phosphate dehydrogenase, G6PD; malic enzyme, ME; and acetyl CoA carboxylase) were detected and did not show any clear change in activity in response to low dietary fat content (Regost et al. 2001). However, the activities of G6PD, ME, FAS, and ACLY in liver of European sea bass were reduced with increasing fat intake, though the TG balance in the liver was not elucidated (Dias et al. 1998). Compared with these two studies above, the work of Lin et al. (1977) in Coho salmon found that the activities of lipogenic enzymes in the liver increased in the LF diet group, but these activities were relatively low and comparable in adipose tissue between different dietary lipid content groups.

**Figure 7:**
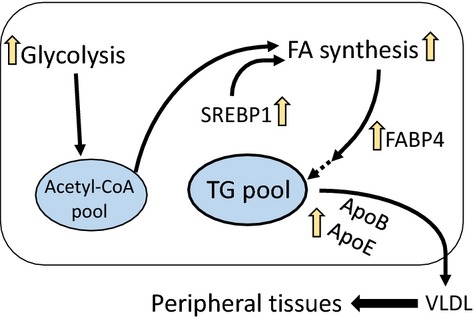
Proposed model of the metabolic adaptation to the low-fat diet in the liver of Nile tilapia. In the liver of LF diet-fed Nile tilapia, accelerated glycolysis provides more substrate – acetyl-CoA for FA and TG synthesis to maintain the content of liver TG. This process is upregulated by SREBP1. The newly synthesized TG can be transported out to other peripheral tissues mediated by increased VLDL consisting of ApoB and ApoE.

In this study, by comparing the systemic metabolic alterations of lipid metabolism in different tissues, liver is verified as the main lipogenic organ in tilapia when dietary lipid intake is low. As a main proof, the significantly higher expression of SREBP1 was seen in the liver of the LF group. SREBP1 plays important roles in lipogenesis, and the proteins encoded by SREBP1 bind to a sequence in the promoter of different genes required for glucose metabolism and lipid production (Eberle et al. 2004). Correspondingly, the SREBP1-targeted lipogenic genes, such as ACCα, ACLY, and FAS, were all upregulated in the liver of the LF group. The metabolic strategy responding to a LF diet in the liver was briefly illustrated (Fig.7). In short, the increased SREBP1-mediated expression of lipogenic genes (ACCα, ACLY and FAS) and intracellular FA transport gene (FABP) promised the FA would be efficiently synthesized and esterified to form TG in hepatocytes. At the same time, high expression of hepatic lipoprotein ApoE in the LF group revealed the possibility that the newly synthesized TG could also be efficiently transported to peripheral tissues for further use. As the final output of lipid homeostasis, the serum TG contents in the LF, MF, and HF groups were comparable. In the case of lipid deficiency, it is possible that higher glycolysis would be stimulated and provide more acetyl-CoA, which will be catalyzed by ACCα to malonyl-CoA for FA synthesis. As proof, our transcriptomic analysis, which was first conducted to examine the effects of dietary lipid levels on physiological response in fish until now, did indicate that glycolysis, hexose metabolic processes, carbohydrate kinase activity, and glucose metabolic processes were included in GO enriched pathways only between LF versus MF and/or LF versus HF, but not between MF versus HF pairs (Table3). Moreover, three direct glycolysis-related limiting enzymes (hexokinase, phosphofructokinase, and pyruvate kinase) tended to highly express in the LF group compared with the MF or HF groups, although there was no significant change for some genes (*P *>* *0.05) (Table4). These data suggested that the glycolysis in the liver of the tilapia fed with the LF diet was highly probable to be stimulated. Furthermore, the higher mRNA expression of ACCα, ACLY, and FAS in the LF group (Fig.2B) indicated that glycolysis-sourced acetyl-CoA would be efficiently transformed to FA. The similar gene expression patterns between MF and HF showed that when the minimal lipid requirement was satisfied, the liver was not sensitive to higher dietary lipid intake. It is of note that the mRNA levels of genes possibly do not fit the real levels or activities of the proteins and metabolites. For example, even if the mRNA levels of lipogenic enzymes were elevated in LF group, the actual increase in FA synthesis might be small if very little substrates were available. However, considering the hepatic TG content was comparable among three groups, and the genes in glycolysis and FA synthesis were upregulated in liver of LF group, our study suggest that in fish, at least in Nile tilapia, the LF diet would accelerate the utilization of carbohydrate to satisfy the physiological requirement of lipid synthesis, and this process is mainly performed in the liver.

**Table 4:** The data of hexokinase, phosphofructokinase, and pyruvate kinase from hepatic transcriptomic assay

Item		LF	MF	HF	LF versus HF	LF versus MF
Gene name	Gene_id	FPKM	FPKM	FPKM	*P*-value	*P*-value
Hexokinase	ENSONIG00000000186	2.27367	0.896305	1.05924	0.03195	0.0112
	ENSONIG00000015846	2.59032	1.73474	2.21806	0.65405	0.24605
	ENSONIG00000016400	2.16001	0.882575	1.1411	0.0923	0.0277
	ENSONIG00000017298	0.065365	0	0	1	1
	ENSONIG00000017024	36.5945	29.0355	28.9209	0.46645	0.47475
Phosphofructokinase	ENSONIG00000009075	4.97003	3.96111	3.73735	0.37995	0.4854
	ENSONIG00000019109	0.236625	0.376833	0.0449021	1	1
	ENSONIG00000003640	3.11663	0.779021	0.930055	0.00585	0.0033
	ENSONIG00000011877	51.6894	32.0229	22.2438	0.01315	0.14715
	ENSONIG00000014930	1.23049	0.648307	0.402476	0.03335	0.15895
Pyruvate kinase	ENSONIG00000002725	7.78104	2.08947	2.80815	0.3122	0.21015
	ENSONIG00000006474	11.233	11.7074	10.9754	0.9407	0.89515

### The strategy of Nile tilapia responding to high lipid intake

It has been known that HF diets cause fat accumulation in the bodies of most animals, including fish. However, different from rodents, lipid storage sites in fish are highly species-specific. Some fish, such as cod, store fat mainly in the liver (Dos Santos et al. 1993), whereas other fish, such as salmon, could store high amounts of fat between muscle fibers (Torstensen et al. 2000). Nevertheless, for most fish, increased lipid contents in the liver and muscle, along with increased mass of visceral adipose tissue, were commonly reported when fish were fed with HF diets (Du et al. 2005; Wang et al. 2005a). In the fish fed with dietary fat, the majority of the intestinal lipoproteins are transported via the lymphatic system before appearing in the circulatory system and being delivered to the liver in fish, like that of mammals (Sheridan 1988). However, the priority of the position of lipid storage has not been fully known, and the systemic strategy of fish responding to HF intake has not been illustrated either. In this study, the mass of adipose tissue of Nile tilapia had a dose-dependent increase with dietary lipid levels; however, the TG contents in the liver and muscle were relatively stable. Therefore, we clarified that adipose tissue is the prior tissue for lipid deposit in Nile tilapia when fed with a HF diet, at least in this 10-week trial.

**Figure 8:**
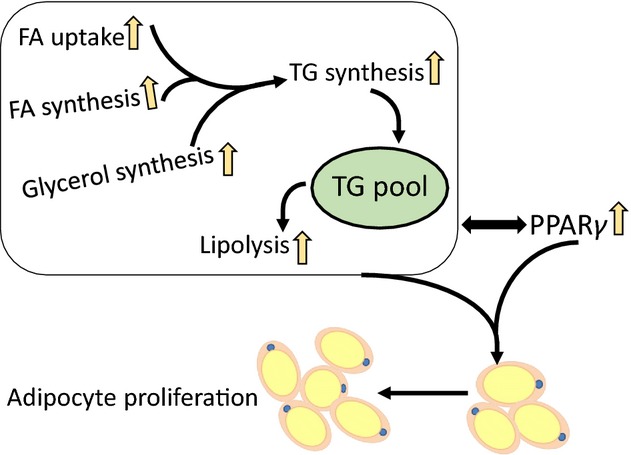
Proposed model of the metabolic adaptation to the high-fat diet in the adipose tissue of Nile tilapia. In the adipocytes of Nile tilapia fed with HF diet, the uptake of diet-sourced FFA, de novo synthesis of FA and glycerol are up-regulated and contribute to increasing TG synthesis. The excess cellular TG is hydrolyzed by accelerated lipolysis and the released FFA, together with the diet-sourced circulating FFA, upregulates PPARγ to trigger the differentiation and proliferation of visceral adipocyte.

In mammals, when energy intake is greater than energy expenditure, adipose tissue swells through increasing the numbers and/or enlarging the size of adipocytes (Rosen and Spiegelman 2006). In this study of Nile tilapia, we first report that the increase in adipocyte numbers is the main metabolic solution to deal with HF intake and this was also confirmed by the enhanced expression of mRNA and protein levels of PPARγ. In mammals, PPARγ functions in adipocyte differentiation and in many cases can convert non-adipose cells to adipocyte-like cells (Rosen et al. 1999; Wu et al. 1999). A number of studies have indicated that HF feeding leads to increased expression of PPARγ and a number of PPARγ-targeted genes involved in adipocyte differentiation and lipid storage (Brun and Spiegelman 1997). Most FA can activate PPARγ (Kliewer et al. 1997) and the highest binding affinity is achieved with 16–20 carbon chain-length FAs (Forman et al. 1997), which are the dominant compounds in the increased FFAs in circulation in the HF feeding state. HF feeding does not only cause the differentiation of adipocytes, but also induce adipogenesis through elevating FA synthesis de novo and TG synthesis (Ilich et al. 2014). The expression of lipogenic genes in adipocytes is controlled by the transcription factors SREBP-1 (Jeon and Osborne 2012). Compared with the upregulated PPARγ in the adipose tissue of Nile tilapia in the HF group, the expression of SREBP1 was not affected by dietary lipid level in this study. However, the mRNA levels of some SREBP1-targeted genes in FA synthesis, such as ACCα and FAS, were highly expressed in HF diet. This discrepancy could be explained by the inconsistency among mRNA levels, protein levels/activities and metabolic flux. The related systemic metabolic mechanism of HF diet-induced proliferation of adipocytes in Nile tilapia is also illustrated (Fig.8). In short, in the state of HF intake in Nile tilapia, diet-sourced FFAs are mainly absorbed by adipocytes, and at the same time, the FA synthesis de novo are also increased. Then glycerol and TG synthesis in adipocytes is elevated to form more TGs, which are stored in lipid droplets. At the same time, lipolysis is also increased to limit the lipid accumulating capacity; thus, the excess accumulated TGs will be hydrolyzed again and released as FFAs from adipocytes. Many studies had indicated that FFAs would upregulate the expression of PPARγ as an endogenous ligand (Spiegelman 1998). Therefore, both the increased lipolysis in adipocytes and the continuous intake of HF diets induce high expression of PPARγ and trigger the process of adipocyte proliferation.

It is of note that the enlargement of adipocytes, which is mainly caused by the increasing size of intracellular lipid droplets, is the normal physiological consequence of rodents in HF diet feeding (Weyer et al. 2000). However, in this study, the adipocyte size and the TG content per unit weight of adipose tissue did not change among three dietary lipid levels in tilapia. Therefore, an interesting question that arises from this study is whether the size of adipocytes would increase with the duration of HF diet feeding, or would the numbers of adipocyte increase continuously. What we can conclude from this study is that the increase in the number of adipocytes is the primary strategy of Nile tilapia, at least in the early stages of the progression of systemic obesity. It should be pointed out that the efficient uptake of high circulating FFAs and transforming these to TGs in specific lipid deposit tissue has been shown to be a protective mechanism against the lipotoxicity of FFAs in many animals responding to temporary HF intake (Boden and Shulman 2002). However, during long-term HF diet feeding, the maintenance of lipid homeostasis will finally fail and result in excess lipid accumulation in other nonlipid-storage tissues, such as skeletal muscle, heart, and liver, along with the high production of many inflammatory and oxidative factors in the endogenous environment (Posey et al. 2009; De La Serre et al. 2010). The similar developing process of HF diet-caused dyslipidemia was also previously reported in fish (Lin et al. 1977; Hemre and Sandnes 1999; Du et al. 2008). Therefore, we suppose that the prolonged duration of HF diet feeding would also cause a series of dyslipidemic symptoms in Nile tilapia, and the lipid metabolism would be largely changed. But it should be pointed out that in that situation, the metabolic changes are mostly pathological consequences rather than physiological adaptations.

### Lipid homeostasis in fish is differently affected by protein, lipid, and carbohydrate intake

In this study in Nile tilapia, the 10-week HF diet feeding did not affect growth and lipid content of the liver, but only increased the mass of adipose tissue. However, in other studies, altered dietary protein and/or carbohydrate for a similar, even shorter duration would significantly affect lipid accumulation in the liver, muscle, and visceral fat mass in fish (Regost et al. 2001; Alam et al. 2009). As in most animals, metabolic energy in fish is available from proteins, lipids, and carbohydrates. Although excess energy is mainly stored in the form of lipid, dietary lipid, protein, and carbohydrate have different physiological roles in lipid homeostasis in fish. Several studies have found that high protein intake normally did not cause severe lipid accumulation in liver, muscle, and visceral adipose tissue in fish (Shyong et al. 1998; Yang et al. 2002, 2003; Alam et al. 2009), which could be because fish preferentially utilize protein as an energy source rather than carbohydrate (Tocher 2003).

In contrast, a number of reports indicated that excess body lipid accumulation is highly correlated to high content of dietary carbohydrate in fish (Tian et al. 2012; Zamora-Sillero et al. 2013; Li et al. 2014). Although the detailed mechanisms are still unknown, some studies have found that the decrease rate of blood insulin level after high carbohydrate feeding in fish is much slower than that in mammals, which is similar to the prediabetic insulin-resistant state in mammals (Warram et al. 1990). Recent studies had indicated that insulin significantly stimulate lipogenesis in rainbow trout (Li et al. 2014). Considering high carbohydrate diet also increases the activities of glycolytic enzymes in some fish species (Pérez-Jiménez et al. 2009), more carbohydrate-sourced acetyl-CoA would be available as the substrate for lipogenesis which is induced by high circulating insulin in the fish fed with high carbohydrate. Although this process has not been fully confirmed, a recent study provided new proofs (Wang 2010). In this study, tilapia were fed with high protein + low carbohydrate diet (HPLC) and low protein + high carbohydrate diet (LPHC), respectively, and body composition and liver transcriptomic assays were performed. The results indicated a LPHC diet significantly increased lipid accumulation in the liver, muscle, and viscera, as compared with an HPLC diet, and this severe lipid accumulation positively correlated with high expression of mRNA of glycolysis and FA synthesis (GCK, ACC, ACLY, FAS). Interestingly, this hepatic gene pattern in the LPHC diet-induced lipid accumulation was very similar with the gene pattern in the LF group in this study. This suggests that either in the case of dietary lipid deficiency or high dietary carbohydrate situations, the stimulated glycolysis may provide the substrates for lipogenesis in tilapia.

In some fish species, HF diet would induce severe fat deposit. This is not only because the excess ingested lipid cannot be efficiently degraded in tissues, but also because the HF diet would impair insulin sensitivity and further impair glucose and lipid homeostasis (Figueiredo-Silva et al. 2012). Comparing the different metabolic responses to the high intake of protein, carbohydrate, and lipid between this study and previous literatures (Brun and Spiegelman 1997; Semple et al. 2006), we demonstrate tilapia could be a species that has the capability to endure a HF diet. The systemic adaptation of lipid metabolism of tilapia responding to a HF diet illustrated by this study could also be regarded as a metabolic model and referenced for other fish species. It should also be mentioned that different ratios of dietary lipid to other nutrients, particularly carbohydrate, would possibly change the metabolic adaptation strategies in tilapia, and this is waiting for further studies.

## Conclusion

High or limited fat intake is commonly seen in farmed or wild fish. However, the detailed metabolic adaptation mechanisms have not been elucidated. This study indicated that LF intake would elevate glycolysis and accelerate biosynthesis of FAs in the liver, which is the primary responding organ to LF diet feeding. In contrast, excess ingested lipid is preferentially stored in adipose tissue through increased capability of FA uptake and TG synthesis. The increase in adipocyte numbers, which is mainly through adipocyte proliferation mediated by PPARγ, but not the enlargement of adipocyte size is the main strategy of Nile tilapia responding to continuous HF diet feeding. This is the first study illuminating the systemic adaptation of lipid metabolism responding to a LF or HF diet in fish, and could be a reference for other fish species.
